# Habitats, Plant Diversity, Morphology, Anatomy, and Molecular Phylogeny of *Xylosalsola chiwensis* (Popov) Akhani & Roalson

**DOI:** 10.3390/plants14152279

**Published:** 2025-07-24

**Authors:** Anastassiya Islamgulova, Bektemir Osmonali, Mikhail Skaptsov, Anastassiya Koltunova, Valeriya Permitina, Azhar Imanalinova

**Affiliations:** 1 Institute of Botany and Phytointroduction, 36D Timiryazev Str., Almaty 050040, Kazakhstan; be96ka_kz@mail.ru (B.O.); v.permitina@mail.ru (V.P.); azhar.imanalinova@gmail.com (A.I.); 2 South Siberian Botanical Garden, Altai State University, Lesosechnaya Str. 25, Barnaul 656906, Russia; mr.skaptsov@mail.ru (M.S.); koltunova.anas@yandex.ru (A.K.)

**Keywords:** *Xylosalsola chiwensis*, DNA molecular analysis, Mangyshlak Peninsula, Ustyurt Plateau, ecological conditions, floristic composition, nature conservation

## Abstract

*Xylosalsola chiwensis* (Popov) Akhani & Roalson is listed in the Red Data Book of Kazakhstan as a rare species with a limited distribution, occurring in small populations in Kazakhstan, Uzbekistan, and Turkmenistan. The aim of this study is to deepen the understanding of the ecological conditions of its habitats, the floristic composition of its associated plant communities, the species’ morphological and anatomical characteristics, and its molecular phylogeny, as well as to identify the main threats to its survival. The ecological conditions of the *X. chiwensis* habitats include coastal sandy plains and the slopes of chinks and denudation plains with gray–brown desert soils and bozyngens on the Mangyshlak Peninsula and the Ustyurt Plateau at altitudes ranging from −3 to 270 m above sea level. The species is capable of surviving in arid conditions (less than 100 mm of annual precipitation) and under extreme temperatures (air temperatures exceeding 45 °C and soil surface temperatures above 65 °C). In *X. chiwensis* communities, we recorded 53 species of vascular plants. Anthropogenic factors associated with livestock grazing, industrial disturbances, and off-road vehicle traffic along an unregulated network of dirt roads have been identified as contributing to population decline and the potential extinction of the species under conditions of unsustainable land use. The morphometric traits of *X. chiwensis* could be used for taxonomic analysis and for identifying diagnostic morphological characteristics to distinguish between species of *Xylosalsola*. The most taxonomically valuable characteristics include the fruit diameter (with wings) and the cone-shaped structure length, as they differ consistently between species and exhibit relatively low variability. Anatomical adaptations to arid conditions were observed, including a well-developed hypodermis, which is indicative of a water-conserving strategy. The moderate photosynthetic activity, reflected by a thinner palisade mesophyll layer, may be associated with reduced photosynthetic intensity, which is compensated for through structural mechanisms for water conservation. The flow cytometry analysis revealed a genome size of 2.483 ± 0.191 pg (2n/4x = 18), and the phylogenetic analysis confirmed the placement of *X. chiwensis* within the tribe Salsoleae of the subfamily Salsoloideae, supporting its taxonomic distinctness. To support the conservation of this rare species, measures are proposed to expand the area of the Ustyurt Nature Reserve through the establishment of cluster sites.

## 1. Introduction

The genus *Salsola* L. was established by Linnaeus in Species Plantarum in 1753 and originally comprised five species. The scope and classification of this heterogeneous and polymorphic genus have been revised multiple times [1]. Until recently, the genus *Salsola* included between 120 and 170 species, the vast majority of which are distributed in the desert regions of Eurasia and Africa [2,3,4,5,6]. In 2007, Akhani H. et al. [7], using molecular genetic methods, published a study in which the genus *Salsola* was split into several genera. One of these was *Xylosalsola* Tzvelev, originally described by N.N. Tzvelev in 1993. The type specimen, previously known as *Salsola arbuscula* Pall., was designated *Xylosalsola arbuscula* (Pall.) Tzvelev. This taxonomic revision gained wider recognition following the work of Akhani H. et al. [7]. Later, in 2015, A.P. Sukhorukov [1] noted that the previously monotypic genus *Xylosalsola* actually included several species.

The members of the genus *Xylosalsola* are subshrubs, shrubs, or low-growing trees with light-gray bark and a highly branched structure. Their leaves, bracts, and perianth segments are covered with short, stiff hairs. Their leaves are alternate, fleshy or filiform, glaucous, almost club-shaped, blunt-tipped, and slightly narrowed or broadened at the base. Their bracteoles are significantly shorter than the leaves and flowers, semicircular, with broad membranous margins and short pointed tips. The perianth segments are broadly lanceolate, narrowly membrane-margined, glabrous or scabrid, and the fruit bears wings below the middle with a loose cone-shaped structure converging above them [2,8,9].

According to Plants of the World Online [10], the genus *Xylosalsola* includes four species: *X. arbuscula*, *X. chiwensis*, *X. richteri* (Moq.) Akhani & Roalson, and *X. paletzkiana* (Litv.) Akhani & Roalson. *X. paletzkiana*, a species of the Southern Turanian region (Afghanistan, Tajikistan, Turkmenistan, and Uzbekistan), occurs on sandy soils and is not found in Kazakhstan. *X. richteri* has a Turanian and Southern Turanian distribution (Kazakhstan, Afghanistan, Iran, Pakistan, Tajikistan, Turkmenistan, and Uzbekistan) and is typically associated with sandy habitats, occurring in the Kyzylkum sands in Kazakhstan. *X. arbuscula* is the most widely distributed species in the genus, with an Irano-Turanian range. In addition to Central Asian countries (Kazakhstan, Kyrgyzstan, Tajikistan, Turkmenistan, Uzbekistan, Afghanistan, Iran, and Pakistan), it is also found in Russia (eastern and southern European parts), Mongolia, and China (Inner Mongolia, north-central China, and Xinjiang).

*X. chiwensis* has a Turanian type of range, is distributed in Kazakhstan and Turkmenistan [10], and has also been reported in Uzbekistan in multiple publications [11,12,13,14]. The species is listed in the Red Data Books of both Kazakhstan and Uzbekistan [11,15].

According to data from iNaturalist and the GBIF database [16,17], the species has been recorded in six localities in Kazakhstan (added by Osmonali B. during the 2024 expedition and by Islamgulova A. in 2025). In the Global Biodiversity Information Facility Secretariat checklist dataset [17], the species is reported in Uzbekistan (Fergana Valley). However, the checklist of the Flora of Protected Natural Areas in the Fergana Valley does not include *X. chiwensis*. This contradicts the distribution indicated in the Red Data Book [11], which states its occurrence in Karakalpakstan on the slopes of the Ustyurt Plateau and in the Kyzylkum sands (Sultan Uveysdag). These discrepancies are not supported by published data [18] and do not correspond to the species’ known ecological preferences, leading us to believe that they are errors. It is likely that mistakes were made in the GBIF database entry.

On the Plantarium website [19], the species was recorded in a single locality in 2024 by V. Kolbintsev in the Ustyurt Nature Reserve (Kazakhstan), in the border region between the Mangyshlak lowlands and the Ustyurt Plateau. The presence of the species in the flora of the reserve is supported by previous publications [20,21]. In 1996, the species was also recorded by I.N. Safronova at two sites in the Mangyshlak lowlands and one site in the southern part of the Ustyurt Plateau, based on records listed under the name of I.N. Safronova, as presented in the Cat-alogue of Rare and Endangered Plant Species of the Mangistau Region [22].

Herbarium specimens of *X. chiwensis* from the collections of the Institute of Botany and Phytointroduction (AA, Almaty, Kazakhstan), Lomonosov Moscow State University (MW; Moscow, Russia), and the Komarov Botanical Institute (LE; Saint Petersburg, Russia) were examined. Based on the studied herbarium collections, a table of specimens was compiled (Table S1). Excluding the records obtained from field research, the herbarium list includes 15 specimens. The herbarium specimens are dated from 1926 to 2015 and 2024.

**Figure 1:**
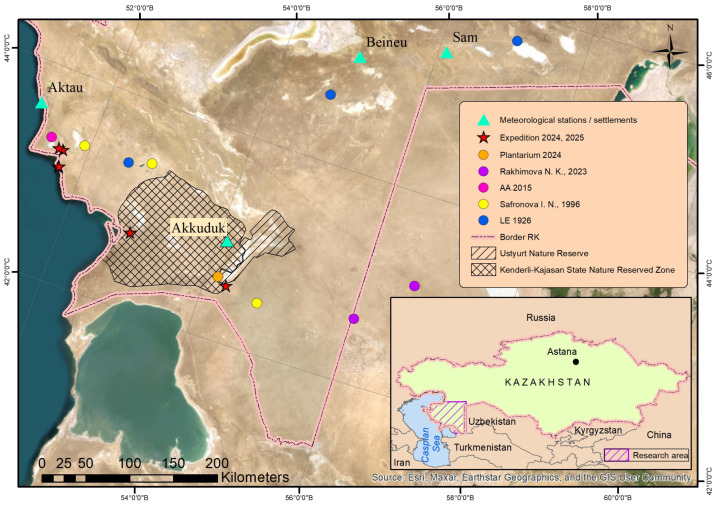
Distribution records of *Xylosalsola chiwensis* in the research area were compiled from fieldwork (2024–2025), herbarium collections, published data by Rakhimova [12], records listed under the name of I.N. Safronova, as presented in the Catalogue of Rare and En-dangered Plant Species of the Mangistau Region [22], and verified online databases.

All available data on the species’ distribution, including field surveys conducted in 2024 and 2025, herbarium specimens, published sources, and verified records from online databases containing georeferenced information, are presented in Figure 1.

Within Kazakhstan, *X. chiwensis* occurs in the territory of the Mangyshlak Peninsula and the Ustyurt Plateau in the Mangystau and Aktobe administrative regions. The area belongs to the North Turanian (southern part of the West-North Turanian subprovince) and Southern Turanian (northern part of the West-Southern Turanian subprovince) botanical provinces of the Irano-Turanian subregion, Saharo-Gobi Desert region [23]. It includes the Mangystau, South Mangystau, North Ustyurt, and South Ustyurt floristic districts [24].

The western Caspian part of the Ustyurt Plateau lies within Kazakhstan, while its eastern (Aral region) part is located in Uzbekistan and Turkmenistan. To the north and northwest, the plateau borders the Caspian Lowland, and to the west it is adjacent to the flat Mangyshlak. The boundary of the plateau is sharply defined by high (150–200 m), deeply dissected cliffs known as “chinks” (a chink is a steep escarpment of Earth’s surface, up to 350 m high, of erosional, denudational, or tectonic origin), which have winding contours. In terms of relief, Ustyurt is classified as an arid denudational mesa plateau. The absolute elevations range from 100 to 120 m in the north to 150 to 300 m in the central and southern parts. The plateau’s relief is characterized as an undulating and gently rolling plain, featuring an alternation of low knolls, gently sloping weakly undulating surfaces, depressions, and hollows. The latter are widespread in the northern part of the plateau and are occupied by solonchaks and sands. Various forms of meso- and microreliefs are well developed on the plateau, especially in the northern and southern parts, including dry valleys, takyrs (a special type of soil consisting of flat, poorly dissected areas of clay in deserts, the formation of which is due to the periodic stagnation of surface waters in conditions of drainless surfaces composed of loams and clays with the participation of salinization and desalinization processes), and depressions, which contribute to the complexity of the soil vegetation cover. The Ustyurt Plateau is characterized by the predominance of gray–brown desert solonetz soils, takyrs, and solonchaks.

The Mangyshlak Plain encompasses the territory situated between the Ustyurt escarpments (chinks), the ridges of the mountainous Mangyshlak, and the coastal strip of the Caspian Lowland. Similar to Ustyurt, the Mangyshlak Plain is an arid denudation table plateau that has been lowered to absolute elevations of 50–150 m, representing the lower tier of the same massif. A distinctive feature of the landscape is the presence of extensive and deep depressions—such as Karagiye (−132 m below sea level) and Kaundy—on the gently undulating surface. These depressions are occupied by solonchaks (salt flats).

Cenopopulations 1–4 are distributed in regions dominated by gray–brown desert solonetzic soils and underdeveloped gray–brown desert soils. The fifth cenopopulation occurs in the Kenderli–Kayasan undulating plain region, characterized by gray–brown desert normal and solonetzic soils in the southern part of the Mangyshlak Plain. The sixth cenopopulation is distributed between the Karynzharyk depression and the western chink of Ustyurt, in the southeastern part of the hilly Mangyshlak, on bozyngens–convex or flat surfaces with highly gypsum-rich gray–brown desert soils [25].

**Figure 2:**
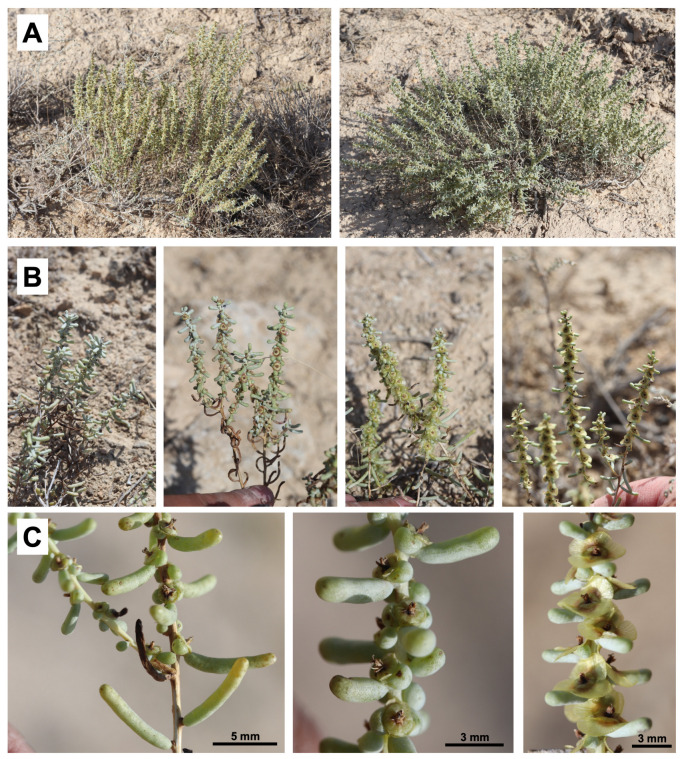
*Xylosalsola chiwensis*: (**A**) General appearance of the plant, (**B**) Branches with leaves and fruits, (**C**) Fragment of a plant.

*X. chiwensis* is a semishrub that is 30–60 cm tall, with strongly branched stems. Its annual shoots are glabrous, smooth, and highly branched. Its leaves are alternate, semi-terete, succulent, and glaucous. The bracts exceed the length of the flowers and bracteoles; the latter are semicircular, glaucous, nearly hemispherical, and narrowly membranous along the margins, while the inflorescence is spike-like. The perianth segments are glabrous and bear wings at the fruiting stage, forming a loose, convergent cone-shaped structure above; the wings are yellowish and semi-transparent; three of them are broader, widely obovate, and the other two are narrow, wing-like appendages [26,27]. The species is a xerophytic succulent, flowering and fruiting from July to September. It reproduces through seeds (Figure 2).

*X. chiwensis* is listed in the Red Data Book of Kazakhstan under Category II. It is a rare species, occurring in small populations and within limited areas. Ecologically, it is associated with saltwort–desert complexes on eroded gray–brown desert soils of marl slopes, typical solonchaks, and solonetzic soils. The main threat to its survival is anthropogenic activity within its habitats, including the construction of industrial facilities and roads, as well as oil and gas extraction [15].

Studies of plant communities in the Mangystau and Aktobe regions conducted in 2024–2025 confirmed that *X. chiwensis* rarely occurs and has a low abundance.

The current state of *X. chiwensis* remains insufficiently studied. Although the species is listed in the Red Data Books of two countries (Kazakhstan and Uzbekistan), it is not included in the IUCN Red List [28] and does not have international conservation status as a rare species, highlighting the relevance and urgency of further research on *X. chiwensis*.

The purpose of this study is to investigate the current status of the rare species *X. chiwensis*. The research objectives were to (1) analyze the natural and climatic conditions of its habitats; (2) analyze the floristic composition of the plant communities; (3) conduct morphological and anatomical studies on the species to determine the phylogenetic position of *X. chiwensis* using molecular genetic analysis; (4) identify threats to the existence of the species; and (5) develop recommendations for the conservation of the species.

## 2. Results

### 2.1. Floristic Composition of Plant Communities

Vegetation surveys on the Mangyshlak Peninsula identified six coenopopulations involving *Xylosalsola chiwensis*. Analysis of the species composition revealed 53 species of vascular plants (Table S2).

The coenoflora of plant communities with *X. chiwensis* on the Mangyshlak Peninsula consisted of 53 vascular plant species, belonging to 39 genera and 15 families. The dominant families were Amaranthaceae (17 species), Poaceae (6), and Asteraceae (5), together accounting for 53% of the coenoflora. The most representative genera were *Anabasis* and *Artemisia*, each comprising four species. The ruderal flora were represented by six species, comprising 11% of the total flora identified: *Ceratocarpus arenarius* L., *Salsola tragus* L., *Alyssum desertorum* Stapf, *Descurainia sophia* (L.) Webb ex Prantl, *Alhagi pseudalhagi* (M.Bieb.) Desv. ex Wangerin, and *Ranunculus testiculatus* Crantz. The contribution of ruderal species in the five studied cenopopulations did not exceed 2% of the total projected cover, while in one cenopopulation, it accounted for 11%, which indicates anthropogenic transformation of certain habitats. This included livestock grazing, traffic on an unregulated network of dirt roads, mole colonies, and locally observed recreation.

**Figure 3:**
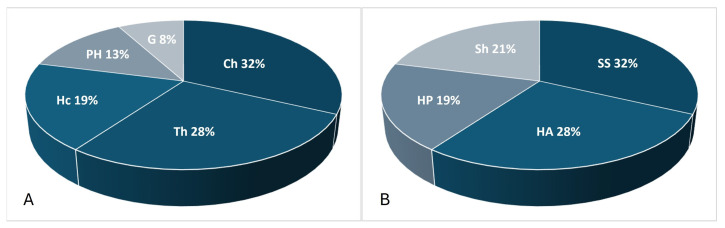
Spectrum of life forms among species co-occurring with *Xylosalsola chiwensis* in studied cenopopulations: (**A**) according to Raunkiaer classification: hemicryptophytes (Hc), geophytes (G), therophytes (Th), chamaephytes (Ch), phanerophytes (Ph); (**B**) according to Serebryakov classification: Tree (Tr); Shrubs and dwarf shrubs (Sh); Semishrubs (SS); Herbaceous forms: perennials (HP), annuals (HA).

In the spectrum of life forms, semishrubs (SS) predominate at 17 species and herbaceous annual (HA) forms at 15 species (Figure 3). The most common group was chamaephytes (Ch), with 17 species. The chamaephytes (Ch) group consists of semishrubs, dwarf shrubs, and dwarf semishrubs with an annual dying-off of the generative shoots. The Ch group is the most characteristic group for the arid zone of Central Asia and is associated with the ability to survive in the dry summer season and droughts [29]. The most common species of this group, with considerable project coverage, were *Artemisia arenaria* DC., *A. gurganica* (Krasch.) Filatova, *A. terrae-albae* Krasch., *Caroxylon gemmascens* (Pall.) Tzvelev, *Anabasis brachiata* Fisch. & C.A.Mey. ex Kar. & Kir., and *Bassia prostrata* (L.) Beck. The next most species-rich group was therophytes (Th), comprising 15 species (28%), and represented primarily by ephemerals, including genera such as *Eremopyrum*, *Alyssum*, and *Ranunculus*, as well as annual saltworts like *Climacoptera crassa* (M.Bieb.) Botsch. and *C. lanata* (Pall.) Botsch.

The coenoflora of plant communities containing *X. chiwensis* in the southern part of the Ustyurt Plateau [12] consisted of 20 species belonging to 18 genera and 13 families. Ruderal species accounted for 10% of the coenoflora.

An analysis of the species composition of *X. chiwensis* coenopopulations from the Mangyshlak Peninsula and the Ustyurt Plateau revealed a Sørensen similarity coefficient of 0.385, indicating a moderate level of similarity, with approximately 24% of the species shared between the two regions. The observed differences in species composition are attributed to climatic conditions and the geographical positions of the regions.

### 2.2. Characteristics of Plant Communities, Abiotic Factors of Habitats, and the Influence of Anthropogenic Factors

**Figure 4:**
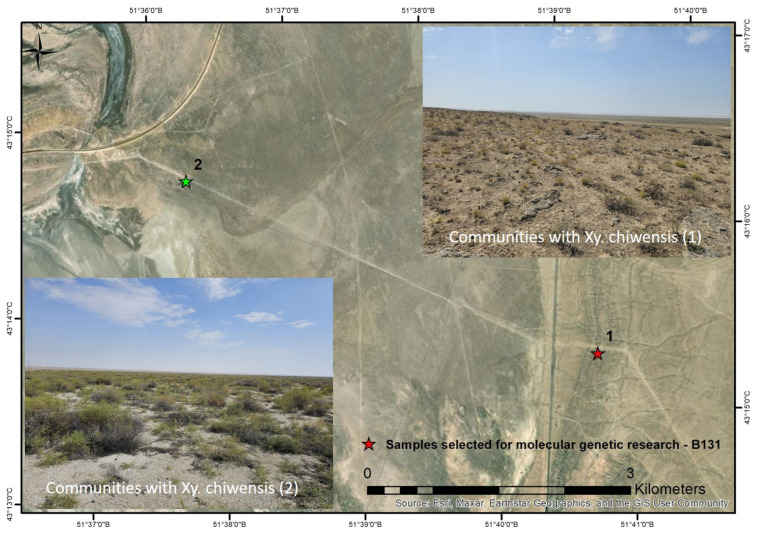
Habitats and spatial distribution of communities 1 and 2 with *Xylosalsola chiwensis*.

Two plant communities differing in ecological conditions were identified between the Karagie and Ashisor depressions (Figure 4). The floristic composition of the communities is presented in Table S1 (No. 1 and 2). *Xylosalsola chiwensis* accounted for no more than 1% of these communities. The distance between the two coenopopulations was greater than 5 km.

The first community (No. 1) was composed of perennial saltwort–sagebrush vegetation, including *Artemisia terrae-albae*, *Oreosalsola arbusculiformis* (Drobow) Sennikov, and *Anabasis brachiata*. It developed on brown, underdeveloped soils of an undulating plain with gentle knolls at an elevation of 24 m above sea level. Bedrock outcrops covered approximately 20% of the surface. The total projective cover (TPC) was 25%. The vegetation showed a low degree of disturbance, primarily from grazing and vehicle traffic. A total of 17 vascular plant species were recorded in the community. The semishrubs (PC: 18%) included *Artemisia terrae-albae*, *A. lercheana* Weber ex Stechm., *Anabasis brachiata*, *A. salsa* (Ledeb.) Benth. ex Volkens, *Nanophyton erinaceum* (Pall.) Bunge, and *Limonium suffruticosum suffruticosum* (L.) Kuntze. The shrubs and dwarf shrubs (PC: 5%) included *Oreosalsola arbusculiformis*, *Convolvulus fruticosus* Pall., *Ephedra distachya* L., and *Atraphaxis spinosa* L. The herbal layer (PC: 2%) consisted of *Poa bulbosa* L., *Stipa arabica* Trin. & Rupr., *Tulipa* spp., *Alyssum desertorum*, *Ranunculus testiculatus*, and *Eremopyrum bonaepartis* (Spreng.) Nevski.

The second community (No. 2) was distributed across a sandy massif of the coastal plain with sor-type depressions, located at an elevation of 3 m below sea level. It was represented by *Alhagi pseudalhagi*–sagebrush (*Artemisia arenaria*) vegetation that developed on stabilized sandy flatlands. The TPC was 35%. A total of 12 vascular plant species were recorded, including the semishrubs (PC: 32%) *Artemisia arenaria*, *A. lercheana*, *Alhagi pseudalhagi*, and *Limonium suffruticosum*, and the shrubs and dwarf shrubs (PC: 1–2%) *Ephedra distachya* and *Atraphaxis replicata* Lam. The herbal layer (PC: 1%) contained *Stipa arabica*, *Salsola tragus*, *Descurainia sophia*, *Echinops albicaulis* Kar. & Kir., and *Zygophyllum turcomanicum* Fisch. ex Boiss. The vegetation cover was in a transitional stage between slight and moderate disturbance due to grazing and anthropogenic impacts (power lines, pipelines, and a railway).

**Figure 5:**
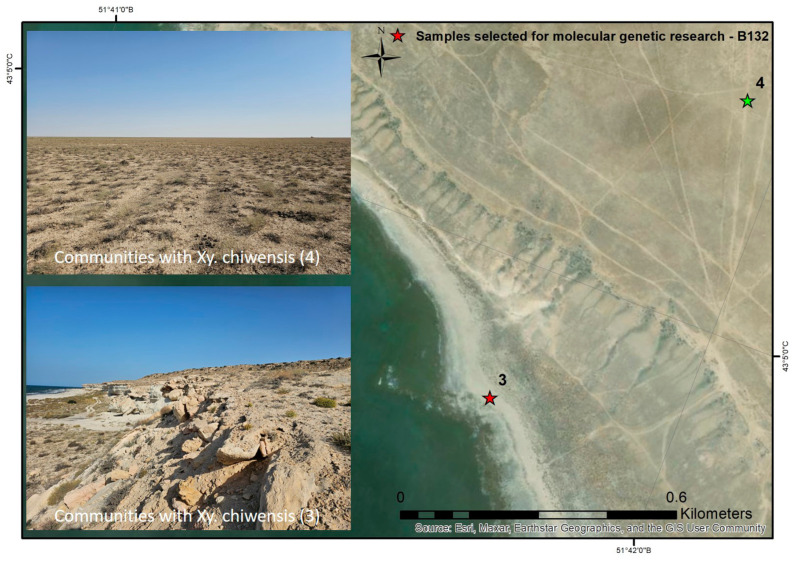
Habitats and spatial distribution of communities 3 and 4 with *Xylosalsola chiwensis*.

The third and fourth plant communities were located approximately 1 km apart near Zhilandy Cape (Figure 5). The third community (No. 3), occupying the western slope of the chink escarpment, was represented by sparse *Bassia prostrata*–sagebrush (*Artemisia gurganica*) vegetation with a total projective cover (TPC) of up to 20%. The coenoflora comprised 15 vascular plant species, including the semishrubs *Artemisia gurganica*, *Bassia prostrata*, *Limonium suffruticosum*, *Caroxylon orientale* (S.G.Gmel.) Tzvelev, *Nanophyton erinaceum*, and *Anabasis truncata* (Schrenk) Bunge, with *X. chiwensis* occurring sporadically; these species dominated the projective cover (PC: 18%). The shrubs and dwarf shrubs (PC: 1%) were *Atraphaxis spinosa* and *Oreosalsola arbusculiformis*. The herbal layer (PC: 1%) contained *Agropyron fragile* (Roth) P.Candargy L., *Poa bulbosa*, *Climacoptera crassa*, and *Eremopyrum distans* (K.Koch) Nevski. The slopes where this vegetation occurred were relatively steep, ranging from 20° to 40°, while the negatively inclined or nearly vertical slopes (80–90°) were almost devoid of plant cover. The soils were eroded brown soils. The plant community was mildly disturbed, with evidence of grazing and dirt roads descending the gentler slopes toward the Caspian Sea.

The fourth community (No. 4) was dominated by sagebrush (*Artemisia gurganica*) vegetation at a TPC of 25%. It was located on a denudation plain of the Mangyshlak region and had developed on brown underdeveloped soils. A total of 11 vascular plant species were recorded. The semishrub layer (PC: 23%) consisted of *Artemisia gurganica*, *A. terrae-albae*, *Anabasis aphylla* L., *Nanophyton erinaceum* (Pall.) Bunge, and *X. chiwensis* (1%). The only shrub or dwarf shrub (PC: 1%) was *Atraphaxis replicata*. The herbal layer (PC: 1%) contained *Ceratocarpus arenarius*, *Eremopyrum orientale* (L.) Jaub. & Spach, *Alyssum desertorum*, *Girgensohnia oppositiflora* (Pall.) Fenzl, and *Onosma staminea* Ledeb. This community was moderately disturbed due to grazing and off-road vehicle traffic. A significant proportion of the sagebrush appeared dry, likely as a consequence of the drought conditions experienced in 2021.

**Figure 6:**
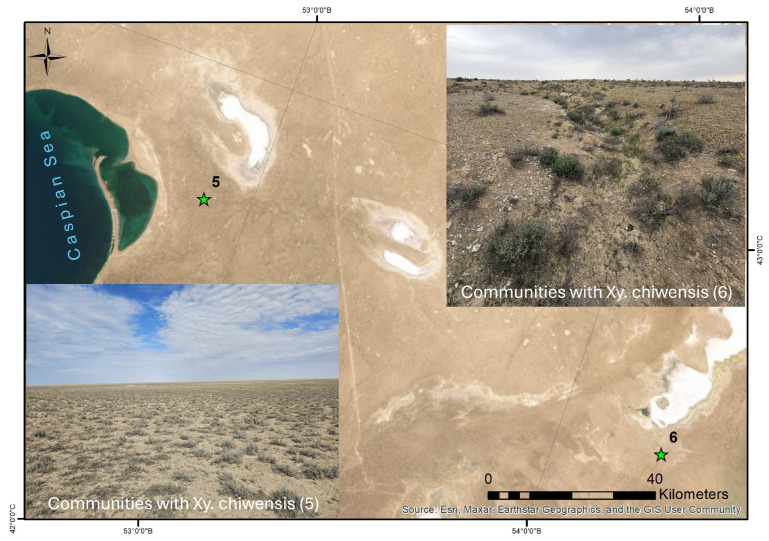
Habitats and spatial distribution of communities 5 and 6 with *Xylosalsola chiwensis*.

The fifth community (No. 5) was located on the Kendirli–Kayasan Plateau, 11 km from the Kendirli Bay and over 110 km from the fourth *X. chiwensis* population (Figure 6). There was a community dominated by *Caroxylon gemmascens* on the gray–brown desert soils on the undulating plain. *Eremopyrum orientale* played a significant role, accounting for up to 5% of the total projective cover (TPC). The TPC was 35%, with 16 species recorded. The semishrubs dominated (PC: 25%) and included the following species: *Caroxylon gemmascens*, *C. orientale*, *Artemisia terrae-albae*, *Anabasis salsa*, *A. brachiata*, *A. truncata*, *Nanophyton erinaceum*, *Limonium suffruticosum*, and *X. chiwensis* (less than 1%). The herbal layer (PC: 5%) consisted of *Eremopyrum orientale*, *Climacoptera lanata*, and *Halothamnus subaphyllus* (C.A.Mey.) Botsch. The shrubs and dwarf shrubs (PC: less than 1%) included *Atraphaxis spinosa*, *Oreosalsola arbusculiformis*, *Convolvulus fruticosus*, and *Ephedra distachya*. The vegetation cover was slightly disturbed, with evidence of grazing, vehicle tracks along dirt roads, and burrowing animal colonies.

The sixth community (No. 6) was found approximately 5 km from the boundaries of two protected areas (the Ustyurt Nature Reserve and Kendirli–Kayasan State Nature Reserve Zone) and over 125 km southeast of the fifth *X. chiwensis* population (Figure 6). Along the runoff depressions on the elongated upland bozyngens, the vegetation was represented by dwarf semishrub groupings, with a total projective cover (TPC) of 10–15%. Twenty-four species were identified, with the semishrub layer (PC: 8%) consisting of *Artemisia gurganica*, *Caroxylon gemmascens*, and *X. chiwensis* (1–2%). The shrubs and dwarf shrubs (PC: 2%) included *Oreosalsola arbusculiformis*, *Convolvulus fruticosus*, and *Ephedra strobilacea* Bunge. The herbal layer (PC: 1–3%) consisted of *Climacoptera lanata*, *Arnebia decumbens* (Vent.) Coss. & Kralik, and *Onosma staminea*, among others, including *Euphorbia sclerocyathium* Korovin & Popov—a species listed in the Red Book of Kazakhstan [15].

The community was slightly disturbed, with the presence of field roads. The coenopopulation of *X. chiwensis*, occurred on soils with a high gypsum content, indicating its broad ecological range.

### 2.3. Flow Cytometry, Morphological–Anatomical and Molecular–Phylogenetic Research

Materials for the flow cytometry, molecular genetic, morphological, and anatomical studies were collected from two populations (Figure 4 and Figure 5). Two herbarium specimens were prepared and deposited in the herbarium collection of the Institute of Botany and Phytointroduction (AA). The specimen numbers are provided in Table S1 (Figure S1).

#### 2.3.1. Morphometric Comparison of *Xylosalsola chiwensis* and *X. arbuscula*

**Figure 7:**
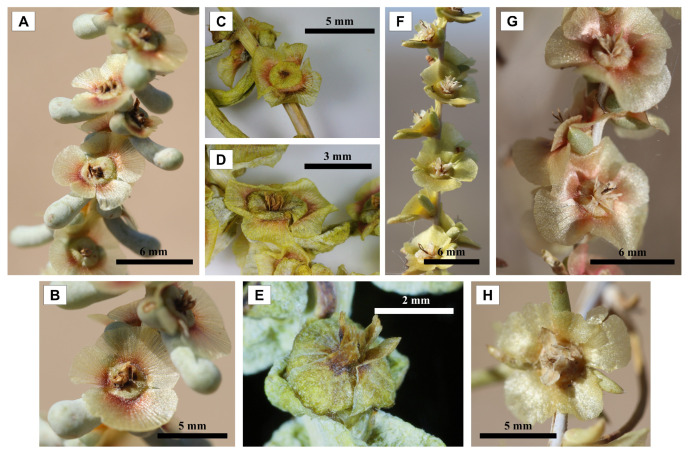
Fruit morphology of two species of the genus *Xylosalsola*. (**A**–**E**) *X. chiwensis*; (**F**–**H**) *X. arbuscula*.

The analysis of the fruits, as the primary morphological characteristic for genus and species identification, was conducted on two species: *Xylosalsola chiwensis* (the study species) and *X. arbuscula* (selected as a comparative specimen) (Figure 7).

The morphological data were compiled into a table (Table 1). The analysis revealed that *X. chiwensis* significantly differs from *X. arbuscula* in all examined morphological traits. The most pronounced differences were observed in the diameter of the fruit (without wings) (5.30 mm in *X. chiwensis* vs. 8.53 mm in *X. arbuscula*) and the length of the cone-shaped structure (1.09 mm vs. 1.90 mm, respectively), as confirmed by high t-statistic values (−19.15 and −13.32) and extremely low *p*-values (less than 0.00000001). These parameters may serve as reliable morphological markers for the identification of *X. chiwensis* under both field and herbarium conditions.

Although the diameter of the fruit (without wings) in *X. chiwensis* is slightly greater than that of *X. arbuscula* (2.81 mm vs. 2.57 mm), and the bract length is shorter (6.07 mm vs. 8.91 mm, respectively), these differences were also statistically significant (*p* < 0.05).

#### 2.3.2. Anatomical Analysis

**Figure 8:**
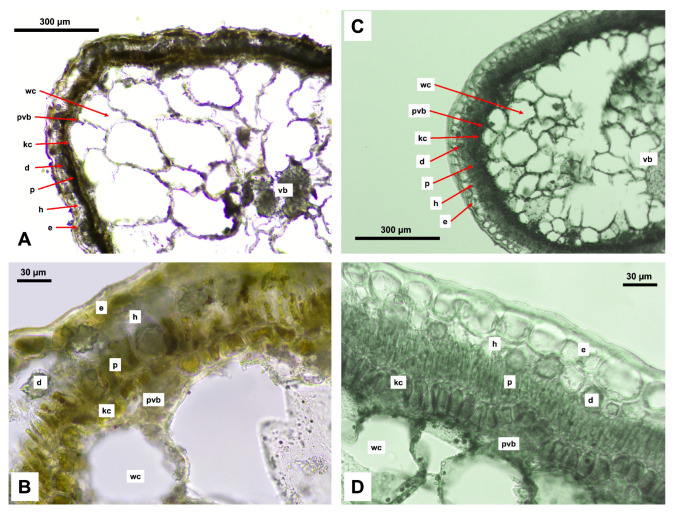
Anatomical cross-sections of a leaf: (**A**,**B**) *Xylosalsola chiwensis*; (**C**,**D**) *X. arbuscula*. e—epidermis; h—hypodermis; p—palisade mesophyll; kc—Kranz cells; d—druse; pvb—peripheral vascular bundle; wc—water-storage cell; vb—vascular bundle.

An anatomical analysis was conducted on the leaves of *Xylosalsola chiwensis* and *X. arbuscula*, as leaf structure remains the most relevant feature in current anatomical studies of the Amaranthaceae family. Both species exhibit the most common leaf anatomical type, classified as the Salsoloid type [30,31,32]. The leaves possess a single-layered palisade parenchyma, and Kranz cells are arranged circumferentially around the leaf. Peripheral vascular bundles are adjacent to the Kranz cells, while the main vascular bundle is located centrally among the water-storage cells. The hypodermis may be present or absent. It is noteworthy that the anatomical section of *X. chiwensis* was prepared from herbarium material collected during the expedition (Figure 8).

The study of the anatomical characteristics of the leaves revealed differences between the two species, reflecting their adaptations to distinct ecological conditions. The numerical data are presented in Table 2.

*X. arbuscula* exhibits a greater thickness of the epidermis, palisade mesophyll, and Kranz cells, which may indicate more effective protection against excessive water loss and higher photosynthetic activity. In contrast, *X. chiwensis* possesses a more developed hypodermis, likely associated with mechanisms for moisture retention.

The enhanced structural protection observed in *X. arbuscula* may be explained by its more arid habitat, whereas *X. chiwensis* employs a different adaptive strategy, balancing photosynthetic activity and water regulation.

These anatomical features not only confirm the ecological adaptability of these species but also serve as additional criteria for their taxonomic differentiation.

#### 2.3.3. Flow Cytometry

The genome size analysis of the two species using flow cytometry revealed that the genome size of *Xylosalsola chiwensis* was 2.483 ± 0.191 pg, while *X. arbuscula* exhibited two genome size variants: 3.250 pg (noted in one sample) and 6.723 ± 0.582 pg (Table 3).

#### 2.3.4. Molecular Genetics

**Figure 9:**
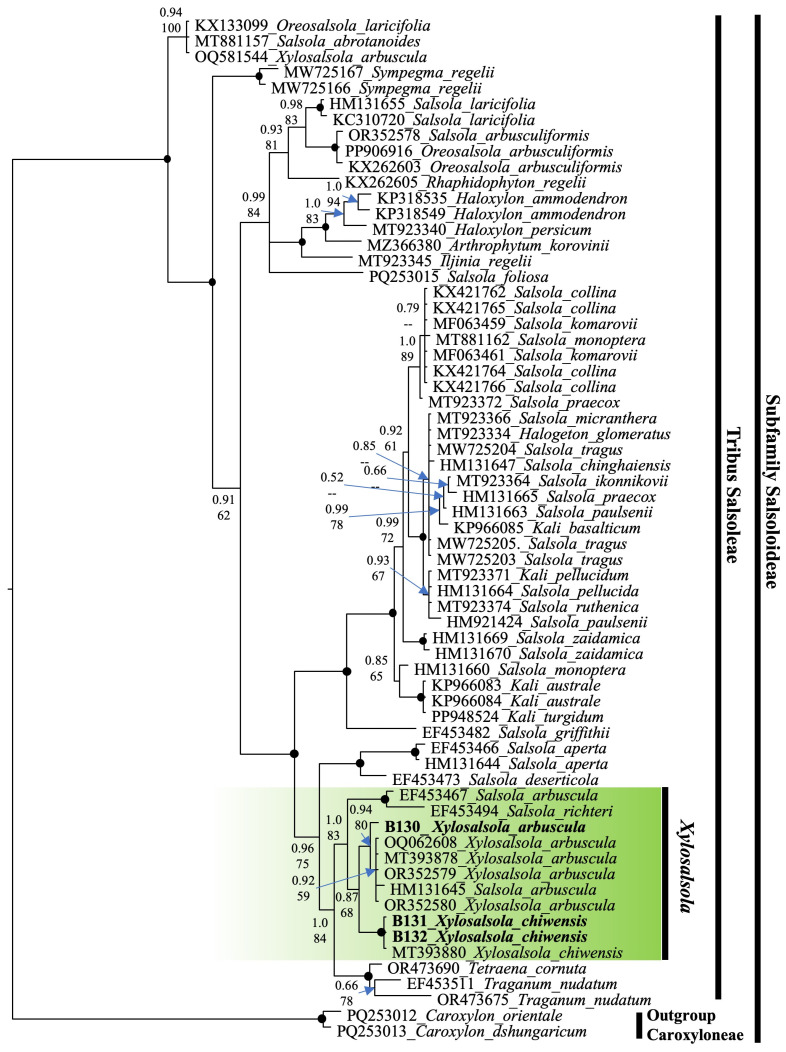
Phylogenetic nrITS tree of the subfamily Salsoloideae. The joint presence of Bayesian with a probability greater than 0.98 and bootstrap support greater than 95% is indicated by a black dot. The genus under study is indicated by a short vertical line and a solid green coloration. Tribes are marked by vertical lines. The accession number of sequences from the NCBI GenBank is given for each species name.

**Figure 10:**
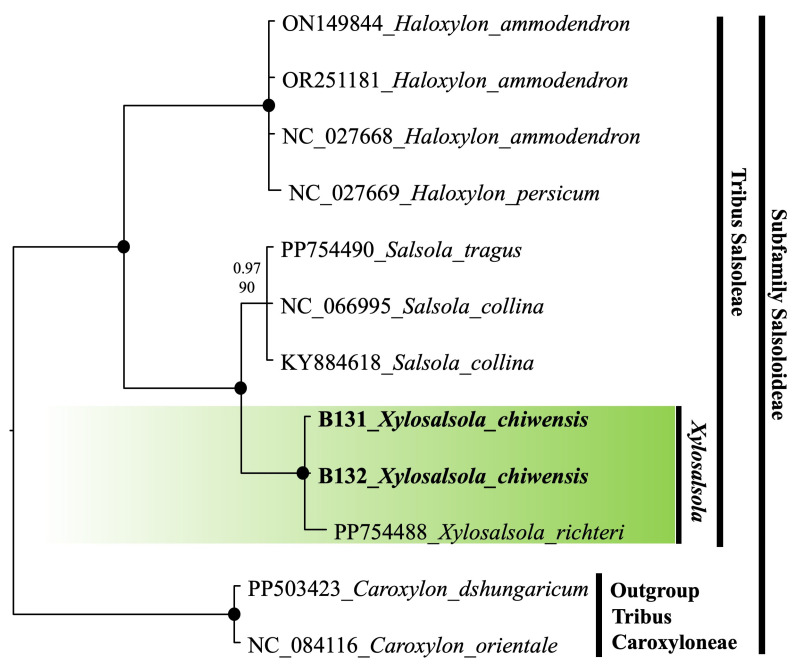
Plastid phylogenetic tree based on the rpsF–rpsR2 region for representatives of the subfamily Salsoloideae (Amaranthaceae). The joint presence of Bayesian with a probability greater than 0.98 and bootstrap support greater than 95% is indicated by a black dot. The bold font indicates the specimens we selected. The genus under study is indicated by a short vertical line and a solid green coloration. Tribes are marked by vertical lines. The accession number of sequences from the NCBI GenBank is given for each species name.

Nuclear (nrITS) and chloroplast (rps16 intron) fragment sequencing was performed on the two species. The ITS sequence phylogenetic tree was constructed using the results from the studied samples, supplemented with sequences from the NCBI database. To compare and confirm the precise phylogenetic placement of *Xylosalsola* species, available ITS sequences from the subfamily Salsoloideae, particularly from the tribe Salsoleae, were obtained from the NCBI database (Figure 9). Species from the tribe Caroxyloneae, the closest relative to the studied genus—especially *Xylosalsola chiwensis*—were used as the outgroup. Our samples are highlighted in bold font in the tree. The color coding indicates the genetic affiliation of the *Xylosalsola* species (Figure 10).

Both the nrITS and rps16 intron fragments of the genus *Xylosalsola* are well resolved in the phylogenetic tree, with clear distinctions between species. It should be noted that there are insufficient chloroplast fragment (rps16 intron) data available in public databases for the studied and closely related species to construct a more detailed phylogenetic tree.

## 3. Discussion

### 3.1. Floristic Diversity

Plant communities involving *Xylosalsola chiwensis* on the Mangyshlak Peninsula are characterized by moderate floristic diversity and a life form spectrum typical of arid ecosystems. The dominance of subshrubs and annuals reflects adaptation to a dry climate, where the main vegetation phase is restricted to a short spring period. A significant proportion of chamaephytes indicates the resilience of these forms to drought and thermal stress, in line with the ecological conditions of Central Asian deserts [29,33].

The low presence of ruderal species in most coenopopulations of *X. chiwensis* suggests the relative integrity of natural vegetation. Increased anthropogenic pressure, associated with uncontrolled grazing, vehicle traffic, industrial infrastructure development, and burrowing animal activity, is evident in moderately disturbed coenopopulations. These factors lead to the localized transformation of the vegetation cover.

Moderate floristic similarity between communities of the Mangyshlak Peninsula and the southern Ustyurt Plateau reflects regional differences in climate, relief, and disturbance levels. Despite the overall arid conditions, variation in species composition highlights the importance of local ecological factors.

The findings emphasize the significance of floristic surveys for assessing community resilience under increasing anthropogenic pressure and climate change.

### 3.2. Ecological Features and Community Structure Involving Xylosalsola chiwensis

*Xylosalsola chiwensis* occurs in various habitats, including denudation plains, sandy flatlands, and undulating terrains with high gypsum content. Although its cover is generally low, the species’ stable occurrence across diverse environments indicates ecological tolerance to temperature extremes, water deficit, and soil salinity.

The structure of *X. chiwensis*-dominated communities varies in terms of total cover and species composition depending on relief, soil characteristics, and disturbance level. Anthropogenic impacts are accompanied by reduced species richness.

The arid climate—characterized by low precipitation, high summer temperatures, and interannual variability—favors xerophytic subshrubs that form stable but sparse phytocoenoses. Climate, edaphic, and anthropogenic factors should be jointly considered when assessing community conditions and planning conservation efforts.

Given the influence of natural and anthropogenic factors on community structure, data on the distribution of rare species are especially valuable, particularly near protected areas where they help define buffer zones crucial for biodiversity maintenance.

Understanding the ecological and coenotic characteristics of *X. chiwensis* provides a basis for further analysis of its biological traits and phylogenetic affiliation. A comprehensive study encompassing cell structure, morpho-anatomical traits, and molecular markers enables deeper insights into its adaptation mechanisms and taxonomic status.

### 3.3. Diagnostic Features of Xylosalsola chiwensis

#### 3.3.1. Fruit Morphology

The results of morphometric analysis support the morphological distinctiveness of *Xylosalsola chiwensis* from its close relative *X. arbuscula*. The most stable and contrasting traits, such as fruit diameter including wings and cone-shaped structure length, exhibit low intraspecific variability and high diagnostic value. The fruits of *X. chiwensis* are characterized by a smaller diameter but greater uniformity, reflecting a narrower ecological niche and specific adaptation to habitat conditions.

High variability in the length of bracteal leaves limits their utility for taxonomic differentiation between closely related species.

These findings confirm the taxonomic distinctiveness of *X. chiwensis* and underscore the need for comprehensive morphometric assessment when differentiating closely related *Xylosalsola* species.

#### 3.3.2. Anatomy

The anatomical features of *Xylosalsola chiwensis* reflect adaptation to arid environments. A well-developed hypodermis and structurally stable epidermis appear to reduce transpiration and help conserve moisture, thereby supporting survival under water-limited conditions. These traits, along with the presence of a Salsoloid-type Kranz anatomy, indicate an efficient water-use strategy under climatic stress.

Compared to *X. arbuscula*, the reduced thickness of the palisade mesophyll and Kranz cells in *X. chiwensis* may reflect a trade-off between photosynthetic intensity and water use efficiency. This balance is crucial for survival on marl slopes, saline depressions, and brown to gray–brown desert soils, where the species commonly occurs.

The identified anatomical traits broaden the understanding of the species’ ecological tolerance and may hold taxonomic relevance. The structural stability of key tissues, including low variability in epidermal thickness, supports their diagnostic value at the species level. The results highlight the importance of an integrated approach combining anatomical and ecological data in studies of adaptation and species differentiation within *Xylosalsola*.

#### 3.3.3. Flow Cytometry

For the first time, the nuclear genome size of *Xylosalsola chiwensis* (2.483 ± 0.191 pg) has been determined, expanding our understanding of the cytogenetic diversity within the genus *Xylosalsola* and confirming its diploid status (2n = 18), which is typical for several members of the subfamily Salsoloideae. The measured genome size falls within the range characteristic of C_3_–C_4_ intermediate or C_4_ species adapted to arid environments, consistent with the observed anatomical and morphological traits and the species’ putative photosynthetic type.

The heterogeneity observed among *X. arbuscula* samples, including possible polyploid forms, highlights intraspecific cytogenetic plasticity and supports previously reported structural variability within the genus. This underscores the value of flow cytometry as a tool for assessing species boundaries and chromosome evolution.

The absence of previously published genome size data for *X. chiwensis* increases the value of the results obtained. However, direct karyological studies are required to definitively verify the ploidy level. These findings open the possibility of including *X. chiwensis* in broader phylogenomic and eco-evolutionary studies within the Amaranthaceae family.

#### 3.3.4. Phylogeny

The obtained results confirmed the reliable identification of *Xylosalsola chiwensis* (Figure 9 and Figure 10). The phylogenetic analysis showed that this species belongs to the tribe Salsoleae within the subfamily Salsoloideae, and representatives of the genus *Xylosalsola* form a clade distinct from closely related species of the genus *Salsola* (Figure 9 and Figure 10).

When considering the phylogenetic tree (Figure 9), it is important to note that the number of sequences available for *Xylosalsola* species in the NCBI database is limited. For *X. chiwensis*, only three sequences are available besides our own samples. For tree construction, only the MT393880 sequence [34] was used; the other sequences (AF318632 and AF318642) [35] were too short (510 bp) and therefore excluded from the analysis.

### 3.4. Threats to Existence and Ways to Conservation

*Xylosalsola chiwensis* is a rare species with a narrow distribution range in Kazakhstan, Uzbekistan, and Turkmenistan. It is listed in the Red Books of Kazakhstan and Uzbekistan. However, the latest edition of the Red Book of Turkmenistan excluded this species [36,37]. Reliable up-to-date information on the distribution of *X. chiwensis* in Turkmenistan is lacking.

Populations of *X. chiwensis* continue to face significant pressure from anthropogenic factors and climate change, which could result in population decline and habitat fragmentation. Therefore, the development and implementation of comprehensive conservation measures aimed at preserving the species in its natural habitats is essential.

The main threats to the species’ survival are the following:

1. Habitat fragmentation and degradation: The expansion of infrastructure (roads, pipelines, and industrial facilities) leads to habitat destruction and fragmentation of populations, especially in the northwestern part of the study region. The construction of new unpaved roads intensifies erosion processes, worsening the conditions for natural population recovery.
2. Livestock grazing: This also commonly occurs in the northwestern part. High grazing pressure causes mechanical damage to plants, reducing their reproductive capacity and altering the species composition of plant communities, thereby limiting the potential for natural regeneration.
3. Climate change: Increasing aridization, reduced precipitation, and rising average annual temperatures may negatively impact population stability, especially at early ontogenetic stages [12].
4. Industrial development: In the northwestern part, oil and gas extraction and mining activities alter hydrological regimes, contaminate soils, and increase dust loads, negatively affecting the physiological condition of plants.

The following conservation and protection measures are recommended:

1. Expansion of protected areas: Inclusion of new *X. chiwensis* habitats into the existing Ustyurt Nature Reserve and Kendirli–Kayasan State Nature Reserve zones and establishment of new cluster sites based on research data. For example, the proposal in [22] to designate a site near the tri-border area (Chink Kaplankyr) is considered unjustified because the chink territory is behind a border fence and not exposed to anthropogenic threats.
2. Limiting anthropogenic pressures: Regulation of livestock grazing in *X. chiwensis* habitats, especially in heavily degraded sites, exerting better control over transport route construction, and developing alternative routes to minimize the impacts on natural ecosystems.
3. Scientific research: Regular monitoring of populations with a focus on the ontogenetic structure and reproductive status.
4. Rehabilitation activities: Cultivation of *X. chiwensis* in the Mangyshlak Experimental Botanical Garden with subsequent reintroduction into natural habitats.
5. Awareness and outreach: Informing local communities about the importance of conserving *X. chiwensis* and natural ecosystems as a whole.

## 4. Materials and Methods

### 4.1. Data Collection

This study was conducted in 2024–2025 on the Mangyshlak Peninsula and the Ustyurt Plateau. Six populations of *Xylosalsola chiwensis* were discovered on the Mangyshlak Peninsula, whereas the species was not found within the Kazakhstani part of the Ustyurt Plateau.

Vegetation surveys were conducted using classical geobotanical fieldwork techniques [38]. Each research plot measured 10 × 10 m. Geographical coordinates were determined with a GPS device, and comprehensive geobotanical descriptions were prepared for each site. These included data on coordinates, landscape characteristics, soil types, hydrological conditions, total projective cover, and the degree of anthropogenic transformation of plant communities.

The complete floristic composition was recorded, and for each species, information was gathered on phenological stage, plant vigor (based on a five-point scale), occurrence (following Bykov’s scale), morphometric features (height, habitus), and abundance (expressed as a percentage according to Drude’s scale) [39]. Vegetation descriptions were documented using standardized vegetation survey forms. Our research was complemented by the floristic list of N. K. Rakhimova et al. [12] for two cenopopulations distributed on the Ustyurt Plateau (Uzbekistan), from which six species were added (Table S2).

The spectrum of life forms of species encountered with *X. chiwensis* in the studied cenopopulations is carried out according to the classification of Serebryakov [40] and Raunkier [41].

Plant species identification was performed using the keys from the nine-volume Flora of Kazakhstan [13,42,43,44,45,46,47,48] and the two-volume Illustrated Guide for Identification of the Plants of Kazakhstan [49,50]. Taxonomic names (species, genera, families) were standardized in accordance with the Plants of the World Online database.

To assess the similarity between the species composition of *X. chiwensis* populations on the Mangyshlak Peninsula and the Ustyurt Plateau, the Sørensen similarity coefficient was applied [27,51].

### 4.2. Climate

The climatic conditions were determined based on climatic normals, with the standard period for calculating these normals being 30 years (1991–2020). The data from four meteorological stations (Akkuduk, Aktau, Beineu, and Sam) located within the range of *X. chiwensis* in Kazakhstan (Figure 1) were provided by Kazhydromet [52].

**Figure 11:**
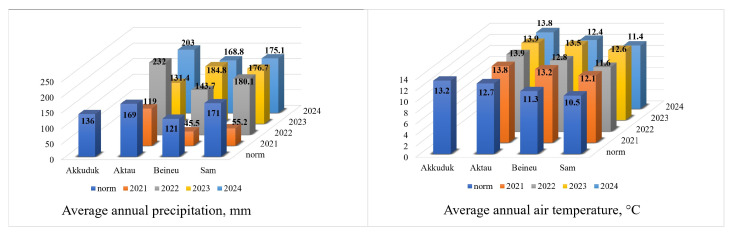
Average annual precipitation and air temperature.

The average annual precipitation norm varied from 171 to 121 mm per year. The data from the past four years (no data available for the Akkuduk station in the registry) were variable (Figure 11), with the precipitation in a dry year (2021) only reaching 32% of the norm, while in a wet year, it exceeded the norm by 37%. The average annual air temperature norm ranged from +10.5 to +13.2 °C. Over the last four years, there has been a trend of an increase in temperature of approximately 1.5 °C on average per year, with minimum and maximum deviations of 1.2 °C and 2.2 °C, respectively.

**Figure 12:**
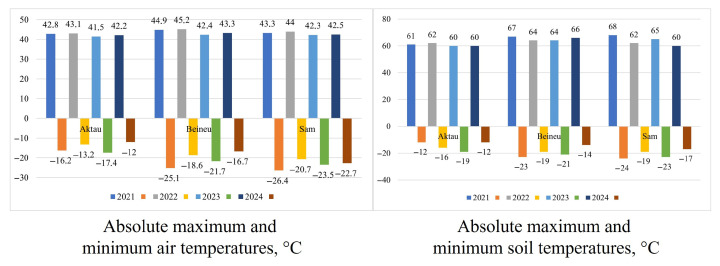
Absolute maximum and minimum air and soil surface temperatures.

The absolute maximum and minimum air temperatures (Figure 12), recorded from 2021 to 2024, show a maximum of 45.2 °C (WS 2022, Beineu station) and a minimum of −26.4 °C (WS 2021, Sam station). The absolute maximum and minimum soil surface temperatures reached 68 °C (WS 2021, Sam station) and −24 °C (WS 2021, Sam station), respectively.

The average monthly temperatures throughout the year, according to the climate normals, ranged from −7.4 to +30.1 °C. It ranged from −7.4 to +1.3 °C in winter, from +2.1 to +21.2 °C in spring, from +23.5 to +30.1 °C in summer, and from +20.8 to +0.8 °C in autumn (Table S3). The average monthly maximum air temperature varied from −3.5 to +4.9 °C in winter, from +7.9 to +28.6 °C in spring, from +29.3 to +37.4 °C in summer, and from +5.8 to +28.6 °C in autumn (Table S4).

The average monthly minimum air temperature ranged from −1.5 to −10.9 °C in winter, from +2.5 to +14.2 °C in spring, from +17.9 to +22.2 °C in summer, and from −3.2 to +15.5 °C in autumn (Table S5).

### 4.3. Morphometric Analysis

Morphometric analysis was conducted on a sample of 30 specimens per species to ensure detailed evaluation and data standardization. The following morphological traits were measured: fruit diameter with wings, fruit diameter without wings, length cone-shaped structure, and bract length. All measurements were performed using LevenhukLite software, version 4.12.28273 (Levenhuk, Inc., Tampa, FL, USA), pre-calibrated with a millimeter scale to ensure measurement precision. The data were statistically processed; mean values and standard errors (SE) were calculated using the Data Analysis Toolpak in Microsoft Excel.

To assess the significance of differences between species, Welch’s *t*-test for independent samples was applied. This method is particularly appropriate when comparing biological objects with heterogeneous variability, as it accounts for unequal variances. The calculations were based on mean values, standard deviations, and a sample size of *n* = 30 for each species, ensuring high reliability of the results. The null hypothesis stated that there were no statistically significant differences between the species, whereas the alternative hypothesis assumed the presence of such differences. Statistical significance was evaluated at the *p* < 0.05 level [53,54,55,56].

#### 4.3.1. Anatomic Method

Anatomical preparations were carried out in accordance with standard histological protocols. Specimens designated for sectioning were initially fixed in 70% ethanol. To facilitate high-quality transverse sectioning, the material was subjected to freezing and subsequently embedded in histological paraffin, poured into specialized molds measuring 15 × 15 mm. Transverse sections were obtained using a rotary semi-automatic microtome (MEDITE M530, Burgdorf, Germany), with a section thickness of 40 µm. Microscopic examination of the anatomical structures was conducted using a digital microscope (Levenhuk Zoom&Joy, Hong Kong, China) equipped with a Levenhuk D740T 5.1 camera. Image acquisition and morphometric measurements were performed using LevenhukLite software, version 4.12.28273 (Levenhuk, Inc., Tampa, FL, USA). Biometric parameters were processed statistically; mean values and standard errors were calculated using the “Data Analysis” function in Microsoft Excel [31,32,57,58,59,60].

#### 4.3.2. Flow Cytometry

The nuclear DNA content was quantified using flow cytometry following propidium iodide (PI) staining. Silica gel–dried leaf tissues were employed as the source of nuclear material. Nuclei were isolated by finely chopping the plant tissue with a sharp razor blade in LB01 buffer supplemented with 50 µg/mL PI, 50 µg/mL RNase [61], 12 mM sodium thiosulfate, and 1% polyvinylpyrrolidone [62]. The resulting nuclear suspension was filtered through a 30 μm nylon mesh to remove cellular debris.

Flow cytometric analysis was performed using a CytoFLEX cytometer (Beckman Coulter, Inc., Brea, CA, USA), and fluorescence histograms were visualized and analyzed using CytExpert software version 2.4 (Beckman Coulter, Inc., Brea, CA, USA). Descriptive statistical analysis, including determining mean values and standard deviations, was carried out using XLStat version 2022.5.1 (Addinsoft, Paris, France). The internal standards included *Pisum sativum* ‘Ctirad’ (2C = 9.09 pg) [62], *Petroselinum crispum* ‘Moss Curled 2’ (2C = 4.50 pg), and *Solanum pseudocapsicum* (2C = 2.835 pg) [63], ensuring the accurate calibration of fluorescence intensities. For flow cytometry and molecular genetic analyses, samples were collected from two distinct populations. As a comparative reference, the type species *X. arbuscula*, upon which the genus is based, was included in the study.

#### 4.3.3. Molecular Genetics Methods

##### Amplification and Sequencing

DNA extraction and amplification were performed using standard molecular protocols at the Laboratory of Bioengineering of the South Siberian Botanical Garden, Altai State University [64]. Total genomic DNA was isolated from silica gel–dried leaf tissue using the DiamondDNA commercial kit (LLC “Altaibiotech”, Barnaul, Russia), following the manufacturer’s instructions.

Two DNA regions were sequenced for phylogenetic analysis: the nuclear ribosomal ITS1–5.8S–ITS2 region and the chloroplast intron rps16. The ITS fragment was amplified using the primers ITS-for (5′-CGTAACAAGGTTTCCGTAG-3′) and ITS-rev (5′-GGAATCCTTGTAAGTTTCTTT-3′) [65]. PCR amplification was conducted in a MyCycler thermal cycler using the following conditions: initial denaturation at 95 °C for 60 s, primer annealing at 57 °C for 30 s, and extension at 72 °C for 30 s over 33 cycles.

The chloroplast rps16 intron was amplified using the primers rpsF (5′-GTGGTAGAAAGCAACGTGCGACTT-3′) and prsR2 (5′-TCGGGATCGAACATCAATTGCAAC-3′) [66]. Amplification reactions were performed in a total volume of 15 μL using a ready-to-use HS-Taq PCR mix (BioMaster, Novosibirsk, Russia) with a final primer concentration of 400 nM. Primers were synthesized by a commercial supplier. The PCR mix consisted of 1 μL of DNA template, 1 μL of each primer, 10 μL of 2× PCR mix, and 8 μL of distilled water.

For ITS amplification, a 20 μL reaction volume was used, including 1 μL of each primer (forward and reverse), 10 μL of ready-to-use PCR mix, and 8 μL of distilled water. Amplified products were verified by electrophoresis in a 1.5% agarose gel stained with ethidium bromide. DNA fragments were visualized under UV light using a Gel IX20 Imager (INTAS, Göttingen, Germany) and documented with a Mitsubishi P93D thermal printer (Mitsubishi Electric Corporation, Tokyo, Japan). Purification of amplification products was carried out using microcolumns (Alove School Supplies Store, China). Sequencing was performed using the Sanger method on an ABI PRISM 3500 XL sequencer (Institute of Chemical Biology and Fundamental Medicine, SB RAS, Novosibirsk, Russia). Sequence chromatograms were manually edited using Chromas Lite 2.1, aligned with ClustalX [67], and manually refined in MEGA11 [68].

##### Phylogenetic Analyses

Separate analyses were conducted for the nuclear ITS and chloroplast rps16 datasets (Table 4) using Fitch parsimony under heuristic search settings in PAUP 4.0b10 [69], employing MULTREES, TBR branch swapping, and 100 replicates of random sequence addition. Indels were treated as missing data. The consistency index (CI) was calculated to assess homoplasy [70]. The most parsimonious trees were summarized using the strict consensus method. Bootstrap support values (BS) were calculated using 1000 pseudoreplicates to evaluate clade support [71]. Bayesian phylogenetic inference was conducted using MrBayes v3.1.23 [72]. The appropriate substitution model was selected based on the Akaike Information Criterion (AIC) using jModelTest2. Two independent analyses were run with four Markov chains for 10 million generations, with tree sampling every 100 generations. The first 25% of sampled trees were discarded as burn-in. The remaining 150,000 trees were pooled to construct a majority-rule consensus tree with posterior probabilities (PP).

To root the phylogenetic trees, species from the tribe Caroxyloneae were used as outgroups, including *Caroxylon orientale* and *C. dzhungaricum* [73].

## 5. Conclusions

This study provides a comprehensive analysis of *Xylosalsola chiwensis*, a rare and vulnerable species endemic to the arid ecosystems of Central Asia. It integrates its ecological characteristics, floristic composition, morphological and anatomical features, genome size data, and phylogenetic placement to contribute to our understanding of the species’ adaptive mechanisms and taxonomic status.

Our findings confirmed the following:

- There is moderate floristic similarity between the populations on the Mangyshlak Peninsula and the Ustyurt Plateau (Sørensen index = 0.385), reflecting the influence of regional environmental factors. These results are consistent with previously reported floristic differences in the area.
- There are pronounced morphological distinctions between *X. chiwensis* and the closely related species *X. arbuscula*, particularly in fruit size and cone-shaped structure length, which serve as important taxonomic characteristics.
- *X. chiwensis* shows anatomical adaptations to arid conditions, including a well-developed hypodermis, thin epidermis, and characteristic mesophyll structure, which align with general patterns of xerophytic adaptation among desert plants.
- The genome size of *X. chiwensis* (2C = 2.483 pg) is reported here for the first time, and the species’ ploidy level was confirmed, which aligns with the polyploid tendencies observed within the genus *Xylosalsola* and the family Amaranthaceae.
- The phylogenetic placement of *X. chiwensis* was confirmed to be within the tribe Salsoleae, distinguishing it as a separate lineage within *Xylosalsola* based on its nrITS and rps16 intron sequences.

A review of the literature and our field observations identified the main threats to *X. chiwensis*: habitat fragmentation and degradation (particularly in the northwestern part of the study region), livestock grazing, climate change, and industrial development. In line with established approaches for the conservation of rare species, we propose the following measures: the expansion of protected areas based on scientific justification of key habitat importance, the regulation of anthropogenic pressures, population monitoring, rehabilitation activities, and raising public awareness regarding the species’ conservation value.

The results significantly enhance our knowledge of *X. chiwensis* and provide a scientific foundation for the development of effective conservation strategies under global change. Future research directions include assessing its genetic diversity and mechanisms of adaptation to extreme arid environments, as well as implementing proposed conservation measures, with a particular emphasis on detailed studies in Turkmenistan, where data on the species’ distribution and population status remain insufficient.

## Figures and Tables

**Table 1:** Comparative morphometric characteristics of fruit structures in *Xylosalsola chiwensis* and *Xylosalsola arbuscula*.

Parameters	*Xylosalsola chiwensis*	*Xylosalsola arbuscula*
Fruit diameter with wings, mm	Mean ± SE: 5.30 ± 0.02 Min/Max: 5.19/5.37 SD: 0.06	Mean ± SE: 8.53 ± 0.17 Min/Max: 7.85/9.53 SD: 0.53
Fruit diameter without wings, mm	Mean ± SE: 2.81 ± 0.09 Min/Max: 2.47/3.18 SD: 0.29	Mean ± SE: 2.57 ± 0.06 Min/Max: 2.29/2.75 SD: 0.19
Cone-shaped structure length, mm	Mean ± SE: 1.09 ± 0.03 Min/Max: 0.90/1.19 SD: 0.09	Mean ± SE: 1.90 ± 0.05 Min/Max: 1.60/2.13 SD: 0.17
Bract length, mm	Mean ± SE: 6.07 ± 0.27 Min/Max: 4.93/8.03 SD: 0.87	Mean ± SE: 8.91 ± 1.13 Min/Max: 5.18/12.94 SD: 3.59

Note: SD—standard deviation.

**Table 2:** Anatomical leaf structure of two species.

Parameter (µm)	*Xylosalsola chiwensis*	*Xylosalsola arbuscula*
Epidermis (E)	20.09 ± 2.24 (15.72–24.40)	35.03 ± 3.29 (30.20–39.00)
Hypodermis (H)	20.96 ± 2.97 (16.94–25.82)	16.31 ± 2.43 (11.60–20.60)
Palisade Mesophyll (P)	29.59 ± 2.88 (23.76–33.19)	33.67 ± 2.98 (30.00–41.50)
Kranz Cells (KC)	17.44 ± 2.97 (13.32–22.24)	21.55 ± 4.28 (14.50–29.00)

Notes: The mean value is given along with the standard deviation (±). The range of values (minimum–maximum) is provided in parentheses.

**Table 3:** DNA content of the studied *Xylosalsola* samples.

Species	Mean 2C ± SD, pg	CV
*Xylosalsola arbuscula*	3.250	-
*Xylosalsola arbuscula*	6.723 ± 0.582	8.65%
*Xylosalsola chiwensis*	2.483 ± 0.191	7.68%

Note: SD—standard deviation; pg—picograms; CV—coefficient of variation.

**Table 4:** Origin and number of sequences used for phylogenetic analyses.

Accession	Name	Coordinates	Voucher	rITS	rps16f-rps16r2
B130	*Xylosalsola arbuscula*	44.79060083 N 63.14563274 E	AA0003576	PV032237	-
B131	*Xylosalsola chiwensis*	43.250147 N 51.671042 E	AA0003564	PV032238	PV036952
B132	*Xylosalsola chiwensis*	43.080778 N 51.696008 E	AA0003563	PV032239	PV036953

## Data Availability

The datasets generated during the study are available from the corresponding authors upon reasonable request.

## Supplementary Materials

The following supporting information can be downloaded at: https://www.mdpi.com/article/10.3390/plants14152279/s1, Figure S1: A—Type specimen (AA); B, C—Specimens collected during this study; Table S1: Herbarium data; Table S2: List of vascular plants with *Xylosalsola chiwensis* described in Kazakhstan and Uzbekistan; Table S3: Average monthly and annual air temperature, °C; Table S4: Average monthly maximum air temperature, °C; Table S5: Average monthly minimum air temperature, °C.
