# Supplementary material for: Habitats, Plant Diversity, Morphology, Anatomy, and Molecular Phylogeny of Xylosalsola chiwensis (Popov) Akhani & Roalson
Source: Plants (Basel). 2025 Jul 24;14(15):2279. doi: 10.3390/plants14152279 (PMC12348769; doi:10.3390/plants14152279)
Supplement: Supplementary file 1 [file plants-14-02279-s001.zip › Table S5. Average monthly minimum air temperature, °C.pdf]

Table S5. Average monthly minimum air temperature, °C

Station	I	II	III	IV	V	VI	VII	VIII	IX	X	XI	XII
Akkuduk	-6,6	-5,5	0,3	7,3	13,9	19,2	22,2	19,9	13,1	5,7	-0,7	-4,9
Aktau	-3,1	-2,7	2,2	8	14,2	19,1	21,4	21	15,5	9,2	2,6	-1,5
Beineu	-9,2	-8,7	-1,4	6,8	14	19,5	22,1	20	12,8	5,1	-1,9	-7,2
Sam	-10,9	-10,3	-2,5	5,9	12,7	17,9	20,5	18,3	11	3,6	-3,2	-8,5
