# Supplementary material for: Habitats, Plant Diversity, Morphology, Anatomy, and Molecular Phylogeny of Xylosalsola chiwensis (Popov) Akhani & Roalson
Source: Plants (Basel). 2025 Jul 24;14(15):2279. doi: 10.3390/plants14152279 (PMC12348769; doi:10.3390/plants14152279)
Supplement: Supplementary file 1 [file plants-14-02279-s001.zip › Table S1. Herbarium data.pdf]

Table S1. Herbarium data

№	Floristic district	Label	Date of collection	Collectors	Herbarium fund or collection
1	Northern Ust-Urt	Adaevsky uyezd, north-west Ust-Urt loamy, northern slope 5 versts to the west of the Tesyk-tas.	24.05.1926	Rusanov F.N.	LE01278642
2	Mangyshlak	Mangyshlak. Karamandybas Depression. Marl Slopes	01.10.1926	Rusanov F.N.	AA0002452 (ТИП)
3	Mangyshlak	Adaevsky uyezd. Western Ust-Urt. Mangyshlak, Karamandybas Depression. Marl Slopes.	01.10.1926	Rusanov F.N.	LE01278643, LE01278645
4	Mangyshlak	Adaevsky uyezd. Eastern Ust-Urt. Toksanbay	06.10.1926	Rozhevits R. Yu., Heinrichson A. O.	LE01278641
5	Mangyshlak	Turkmenistan, northern shore of the bay.<br>Uzbekistan, Karabura, loamy steppe.<br>Kazakhstan, between Sartasam and Chagaly.	29.06.1932	Lebedinova S.	LE01278650
6	Mangyshlak	Southern Mangyshlak, 15 km north of the Karabugauchka Sartas oil field.	30.07. 1934	Mishchevin Ts.	LE01278648
7	Mangyshlak	Southern Mangyshlak. Closer to the north of Sartas.	30.07.1934	Mishchevin Ts.	LE01278651
8	Mangyshlak	Mangyshlak, 18 km east of Eraliyevo. On limestone debris along the western escarpment of the Kara-Chie Depression.	08.10.1944	Rodin L. E.	LE01278649
9	Mangyshlak	Mangyshlak, Karachiv (Bayyr) Depression. On limestone cliffs and in crevices.	17.10.1944	-	LE01278644
10	South Ust-Urt	Ust-Urt, KoniYaz-Kopka locality, southern *Nanophyton*- sagebrush-*Anabasis* complex	25.08.1949	Grubov V.I.	AA
11	Mangyshlak	Mangyshlak. 3–5 km northwest of the Ashi-Bas well. On dry solonchaks among limestone outcrops.	02.07.1953	Grubov V.I.	AA
12	Mangyshlak	Aero-geological expedition No. 11, southeastern Ustyurt, 9 km east of the Mingali well.	18.08.1959	Viktorov S.	MW0823928, MW0823929
13	Mangyshlak	Kazakh part of Ustyurt. Salt lake Barsakelmas. Upper reaches on limestones.	15.10.1973	Fatb.	LE01278645
14	Mangyshlak	Along the ravines on the clayey soil of Ustyurt and on the edge of the plateau near the Chibinly locality.	-	Korovin A.	LE01278647
15	Mangyshlak	Mangystau Region, Karakiya District. N 43°32.294' E 051°47.319'. Elevation -117 m. Karagiye Depression. Hilly plain, clayey soils.	17.07.2015	Imanbayeva A. A.	AA
16	Mangistau	Mangystau Region, Karakiya District. 43.250147° N, 51.671042° E.	02.09.2024	Islamgulova A.F., Osmonali B.B.	AA0003564
17	Mangistau	Mangystau Region, Karakiya District, Ashisor Depression. 43.251325° N, 51.612481° E.	02.09.2024	Islamgulova A.F., Osmonali B.B.	A point recorded *

18	Mangistau	Mangystau Region, Karakiya District. 43.087544°N, 51.699353° E.	03.09.2024	Islamgulova A.F., Osmonali B.B.	A point recorded *
19	Mangistau	Mangystau Region, Karakiya District. 43.080778° N, 51.696008° E.	03.09.2024	Islamgulova A.F., Osmonali B.B.	AA0003564
20	South Mangystau	Mangystau Region, Karakiya District. 42.731517° N, 52.873381° E.	07.09.2024	Islamgulova A.F., Osmonali B.B.	A point recorded *

* Herbarium materials were collected only from two of the localities marked by us.
** Geographical names are written according to the sources.
