# Supplementary material for: Habitats, Plant Diversity, Morphology, Anatomy, and Molecular Phylogeny of Xylosalsola chiwensis (Popov) Akhani & Roalson
Source: Plants (Basel). 2025 Jul 24;14(15):2279. doi: 10.3390/plants14152279 (PMC12348769; doi:10.3390/plants14152279)
Supplement: Supplementary file 1 [file plants-14-02279-s001.zip › Table S2. List of vascular plants with Xylosalsola chiwensis described in Kazakhstan and Uzbekistan.pdf]

Table S2. List of vascular plants with *Xylosalsola chiwensis* described in Kazakhstan and Uzbekistan.

**Families / Species**	Life forms		Kazakhstan Mangyshlak Peninsula						Uzbekistan Ustyurt plateau	
	*	**	1	2	3	4	5	6	1	2
			Amaranthaceae Juss.							
1. *Anabasis aphylla* L.	Ch	SS	-	-	-	1	-	-	-	-
2. *Anabasis brachiata* Fisch. & C.A.Mey. ex Kar. & Kir.	Ch	SS	3	-	-	-	+	-	-	+
3. *Anabasis salsa* (Ledeb.) Benth. ex Volkens	Ch	SS	1	-	+	-	1	-	-	-
4. *Anabasis truncata* (Schrenk) Bunge	Ch	SS	-	-	+	-	+	-	-	-
5. *Bassia prostrata* (L.) Beck	Ch	SS	-	-	4	-	-	-	-	-
6. *Caroxylon gemmascens* (Pall.) Tzvelev	Ch	SS	-	-	-	-	25	2-3	-	-
7. *Caroxylon orientale* (S.G.Gmel.) Tzvelev	Ch	SS	-	-	1	-	1	-	-	-
8. *Ceratocarpus arenarius* L.	Th	HA	-	-	-	1	-	-	-	+
9. *Climacoptera crassa* (M.Bieb.) Botsch.	Th	HA	-	-	+	-	-	-	-	-
10. *Climacoptera lanata* (Pall.) Botsch.	Th	HA	-	-	-	-	+	+	-	-
11. *Girgensohnia oppositiflora* (Pall.) Fenzl	Th	HA	-	-	-	+	-	-	-	-
12. *Halothamnus subaphyllus* (C.A.Mey.) Botsch.	Ch	SS	-	-	-	-	+	-	-	-
13. *Haloxylon ammodendron* (C.A.Mey.) Bunge ex Fenzl	Ph	Tr	-	-	-	-	-	-	-	1
14. *Kalidium foliatum* (Pall.) Moq.	Ph	Sh	-	-	-	-	-	+	-	-
15. *Nanophyton erinaceum* (Pall.) Bunge	Ch	SS	1	-	1	1	1	-	-	-
16. *Oreosalsola arbusculiformis* (Drobow) Sennikov	Ph	Sh	4	-	+	-	+	+	8	+
17. *Salsola tragus* L.	Th	HA	-	1	-	-	-	-	-	-
18. *Xylosalsola chiwensis* (Popov) Akhani & Roalson	Ch	SS	1	1	+	1	+	1-2	+	1
			Apiaceae Lindl.							
19. *Zosima absinthiifolia* (Vent.) Link	Hc	HP	-	-	-	-	-	-	+	-
			Asteraceae Bercht. & J.Presl							
20. *Artemisia terrae-albae* Krasch.	Ch	SS	10-15	-	-	1	1	-	-	20
21. *Artemisia arenaria* DC.	Ch	SS	-	15-20	-	-	-	-	-	-
22. *Artemisia diffusa* Krasch. ex Poljakov	Ch	SS	-	-	-	-	-	-	5	-
23. *Artemisia gurganica* (Krasch.) Filatova	Ch	SS	-	-	10	20	-	3-5	-	-
24. *Artemisia lercheana* Weber ex Stechm.	Ch	SS	2	2	-	-	-	-	-	-
25. *Echinops albicaulis* Kar. & Kir.	Hc	HP	-	+	-	-	-	-	-	-
			Berberidaceae Juss.							
26. *Leontice incerta* Pall.	Hc	HP	-	-	-	-	-	-	+	-
			Boraginaceae Juss.							

Header spans: **Distribution, projective cover %** covers all columns after Families / Species.

27. *Arnebia decumbens* (Vent.) Coss. & Kralik	Th	HA	-	-	-	-	-	+	-	-
28. *Onosma staminea* Ledeb.	Hc	HP	-	-	-	+	-	+	+	-
29. *Pseudolappula sinaica* (A.DC.) Khoshsokhan, Sherafati & Kaz.Osaloo	Th	HA	-	-	-	-	-	+	-	-
Brassicaceae Burnett										
30. *Alyssum desertorum* Stapf	Th	HA	+	-	-	+	-	-	-	-
31. *Descurainia sophia* (L.) Webb ex Prantl	Th	HA	-	+	-	-	-	-	-	-
32. *Sisymbrium subspinescens* Bunge	G	HP	-	-	-	-	-	+	-	-
33. *Strigosella stenopetala* (Fisch. & C.A.Mey.) Botsch.	Th	HA	-	-	-	-	-	+	-	-
Caryophyllaceae Juss.										
34. *Gypsophila diffusa* Fisch. & C.A.Mey. ex Rupr.	G	HP	-	-	-	-	-	+	-	-
Convolvulaceae Juss.										
35. *Convolvulus fruticosus* Pall.	Ph	Sh	1	-	-	-	+	+	4	-
Ephedraceae Dumort.										
36. *Ephedra distachya* L.	Ph	Sh	1	1	-	-	+	-	-	-
37. *Ephedra strobilacea* Bunge	Ph	Sh	-	-	-	-	-	+	-	-
Euphorbiaceae Juss.										
38. *Euphorbia inderiensis* Less. ex Kar. & Kir.	Th	HA	-	-	-	-	-	+	-	-
39. *Euphorbia sclerocyathium* Korovin & Popov	Hc	HP	-	-	-	-	-	+	-	+
Fabaceae Lindl.										
40. *Alhagi pseudalhagi* (M.Bieb.) Desv. ex Wangerin	Ch	SS	-	10	+	-	-	-	-	-
41. *Astragalus ammodendron* Bunge	Ph	Sh	-	-	-	-	-	+	1	-
42. *Caragana grandiflora* DC.	Ph	Sh	-	-	-	-	-	+	-	-
Liliaceae Juss.										
43. *Tulipa* spp.	G	HP	+	-	-	-	-	-	-	-
44. *Gagea kunawurensis* (Royle) Greuter	G	HP	-	-	-	-	-	+	-	-
Plumbaginaceae Juss.										
45. *Limonium suffruticosum* (L.) Kuntze	Ch	SS	1	1	2	-	1	+	1	-
Poaceae Barnhart										
46. *Agropyron fragile* (Roth) P.Candargy L.	Hc	HP	-	-	1	-	-	-	-	-
47. *Eremopyrum bonaepartis* (Spreng.) Nevski	Th	HA	+	-	-	-	-	-	-	+
48. *Eremopyrum distans* (K.Koch) Nevski	Th	HA	-	-	+	-	-	-	-	-
49. *Eremopyrum orientale* (L.) Jaub. & Spach	Th	HA	-	-	-	+	5	-	+	-
50. *Poa bulbosa* L.	Hc	HP	2	-	+	-	-	+	-	-
51. *Stipa arabica* Trin. & Rupr.	Hc	HP	1	1	-	-	-	-	-	-
52. *Stipa caucasica* Schmalh.	Hc	HP	-	-	-	-	-	-	-	+
Polygonaceae Juss.										
53. *Atraphaxis replicata* Lam.	Ph	Sh	-	+	-	1	-	-	-	-
54.*Atraphaxis spinosa* L.	Ph	Sh	1	-	1	-	1	-	-	-
55. *Calligonum leucocladum* (Schrenk) Bunge	Ph	Sh	-	-	-	-	-	+	-	3
Ranunculaceae Juss.										
56. *Ranunculus falcatus* L.	Th	HA	-	-	-	-	-	-	+	+
57. *Ranunculus testiculatus* Crantz	Th	HA	1	-	-	-	-	-	-	-

Rutaceae Juss.										
58. *Haplophyllum obtusifolium* (Ledeb. ex Eichw.) Ledeb.	Ch	SS	-	-	-	-	-	+	+	-
Zygophyllaceae R.Br.										
59. *Zygophyllum turcomanicum* Fisch. ex Boiss.	Hc	HP	-	+	-	-	-	+	-	-

*Life forms according to Raunkiaer classification: hemicryptophytes (Hc), geophytes (G), therophytes (Th), chamaephytes (Ch), phanerophytes (Ph).
** Life forms according to Serebryakov classification: Tree (Tr); Shrubs and dwarf shrubs (Sh); Semishrubs (SS); Herbaceous forms: perennials (HP), annuals (HA).
