# Supplementary material for: Habitats, Plant Diversity, Morphology, Anatomy, and Molecular Phylogeny of Xylosalsola chiwensis (Popov) Akhani & Roalson
Source: Plants (Basel). 2025 Jul 24;14(15):2279. doi: 10.3390/plants14152279 (PMC12348769; doi:10.3390/plants14152279)
Supplement: Supplementary file 1 [file plants-14-02279-s001.zip › Table S3. Average monthly and annual air temperature, °C.pdf]

Table S3. Average monthly and annual air temperature, °C

Station	I	II	III	IV	V	VI	VII	VIII	IX	X	XI	XII	Year
Akkuduk	-2,9	-1,2	5,7	13,7	21,2	27,4	30,1	28,3	20,8	12,4	3,8	-1,3	13,2
Aktau	-0,3	0,6	5,8	11,7	18,2	23,5	26,1	25,7	20,3	13,5	5,9	1,3	12,7
Beineu	-6,2	-5,1	2,9	12,7	20,5	26,5	28,9	27,2	19,5	10,9	1,7	-4,2	11,3
Sam	-7,4	-6,2	2,1	12,2	20	25,8	28,4	26,4	18,6	10	0,8	-5,1	10,5
