# Supplementary material for: Habitats, Plant Diversity, Morphology, Anatomy, and Molecular Phylogeny of Xylosalsola chiwensis (Popov) Akhani & Roalson
Source: Plants (Basel). 2025 Jul 24;14(15):2279. doi: 10.3390/plants14152279 (PMC12348769; doi:10.3390/plants14152279)
Supplement: Supplementary file 1 [file plants-14-02279-s001.zip › Table S4. Average monthly maximum air temperature, °C.pdf]

Table S4. Average monthly maximum air temperature, °C

Station	I	II	III	IV	V	VI	VII	VIII	IX	X	XI	XII
Akkuduk	1,2	4	12,3	20,9	28,6	34,8	37,4	35,9	28,6	19,8	9,1	3
Aktau	3,3	4,9	10,5	16,7	23,5	29,3	32,1	31,7	26,1	18,8	10,3	4,9
Beineu	-2,8	-0,7	8,5	19,3	27,2	33,1	35,4	34	26,5	17,6	6,3	-0,7
Sam	-3,5	-1,5	7,9	19,1	27,1	33	35,4	33,8	26,3	17,1	5,8	-1,3
